# Preparation of Temperature-Activated Nanomaterial-Enhanced Phase Transition Emulsion and Study on Self-Generating Plugging Particles

**DOI:** 10.3390/nano15221715

**Published:** 2025-11-13

**Authors:** Jiaqin Wang, Dan Bao, Yanjie Yang, Zhipeng Miao, Mingzhong Li, Yangyang Qi, Biao Wang, Taosong Liang, Peng Zhang

**Affiliations:** 1 School of Chemistry and Chemical Engineering, Chongqing University of Science & Technology, Chongqing 401331, China; 2022205018@cqust.edu.cn (J.W.); 2024205057@cqust.edu.cn (Y.Y.); 2024205045@cqust.edu.cn (B.W.); 2024205082@cqust.edu.cn (T.L.); 2013024@cqust.edu.cn (P.Z.); 2 Shaanxi Province Key Laboratory of Environmental Pollution Control and Reservoir Protection Technology of Oilfields, Xi’an Shiyou University, Xi’an 710065, China; kjcmzp@xsyu.edu.cn; 3 Shaanxi University Engineering Research Center of Oil and Gas Field Chemistry, Xi’an Shiyou University, Xi’an 710065, China; 4 Technology Service Company, Sinopec Huabei Oilfield Service Corporation, Zhengzhou 450006, China; limingzhong.oshb@sinopec.com (M.L.); qiyangyang.oshb@sinopec.com (Y.Q.)

**Keywords:** lost circulation, nanomaterial, liquid–solid phase transition, adaptive plugging, self-generated particles

## Abstract

Fractured lost circulation remains a major drilling challenge due to low compatibility between conventional plugging materials and fractures. By utilizing thermosetting resin emulsification and high-temperature crosslinking coalescence, this study developed a temperature-activated nanomaterial enhanced liquid–solid phase transition plugging emulsion. The system adapts to varying fracture apertures, forming plugging particles with a broad size distribution and high strength upon thermal activation. The structural characteristics, mechanical properties, and fracture-plugging performance of the plugging particles were systematically investigated. Results demonstrate that the optimized system, comprising 8 wt.% emulsifier, 0.16 wt.% dispersant, 0.4 wt.% crosslinker, 0.4 wt.% viscosifier, 70 wt.% distilled water, and 2 wt.% nano-silica (all percentages relative to epoxy resin content), can produce particles with a size of 1–5 mm at formation temperatures of 80–120 °C. After 16 h of thermal aging at 180 °C, the particles exhibited excellent thermal stability and compressive strength, with D(90) degradation rates of 3.07–5.41%, and mass loss of 0.63–3.40% under 60 MPa. The system exhibits excellent injectability and drilling fluid compatibility, forming rough-surfaced particles for stable bridging. Microscopic analysis confirmed full curing in 140–180 min. Notably, it sealed 1–5 mm fractures with 10 MPa pressure, enabling adaptive plugging for unknown fracture apertures.

## 1. Introduction

Lost circulation is the phenomenon in which a large amount of drilling fluids loss into the formation during drilling [1,2]. Drilling fluid loss IS one of the major challenges faced in current drilling operation and an important factor restricting safe drilling. Lost circulation will consume a lot of drilling fluid and prolong the drilling time [3]. During drilling, a large amount of drilling fluid is lost into the formation. If not properly addressed, it can lead to well collapse, blowouts, pipe sticking, and even borehole abandonment, resulting in major engineering accidents [4,5,6]. Lost circulation seriously restricts the drilling speed, increases the non-production time, and causes significant economic losses [7]. According to statistics, the occurrence rate of lost circulation accounts for about 20–25% of the total number of drilling wells in the world, and the annual cost of plugging is as high as 40 × 10^8^ USD [8]. With the expansion of oil and gas exploration and development to deep–ultra-deep and unconventional oil and gas fields, drilling engineering is facing many limitations and technical bottlenecks. Fractured formation leakage is one of the complex drilling accidents that are difficult to control [9,10,11], and it is also a bottleneck technical problem that restricts the efficient drilling of shale gas [12], which seriously affects the progress of drilling engineering and brings great risks and challenges to drilling operations [13,14].

The plugging material is the key to leakage prevention and plugging [15]. The conventional plugging materials are mainly composed of rigid particles, elastic particles, and fibers [16]. When the conventional bridging plugging materials are used to deal with complex leakage, there are still limitations: it is difficult to grasp the matching between the particle size of the plugging material and the formation fracture, and the pressure bearing capacity of the plugging slurry is insufficient [17], so the success rate of one-time plugging is low [18,19]. After the completion of plugging with cement slurry and chemical consolidation materials, it is necessary to drill and plug again to increase the non-production operation time. Although the gel plugging material has good self-adaptability and matches well with the fracture size [20], it still has low strength and insufficient temperature resistance.

The traditional idea of bridging plugging material is to add the bridging plugging material to the drilling fluid on the ground and then pump it into the lost circulation zone. In this paper, the idea is to inject the liquid phase plugging emulsion, which is easily enters the unknown fractures with different openings and generates bridging particles with different particle sizes in the fractures, while the particles in the wellbore can be pumped out without drilling plugs. In recent years, in the field of oil and gas, scholars at home and abroad have carried out research on high-molecular polymer material [21,22], supramolecular assembly systems, self-assembly material [23], phase transition fracturing systems, self-growing gel plugging emulsion, in situ autogenetic particle profile control systems, self-consolidation resin plugging emulsion, and phase-change materials [24]. Kuroiwa et al. [25] discovered a class of thermochromic small-molecule organogold compounds, which are different from conventional small-molecule organogels. The gels are formed by introducing an ether bond into the alkyl chain of a divalent cobalt complex of 4-alkyl-1,2,4-triazole, and then changing from a light pink solution to a blue gel phase under chloroform heating conditions. The thermochromic transition is completely reversible. Therefore, it is widely used in the design of thermal response and self-assembled molecular wires. Saudi Aramco mixes resin, curing agent, surfactant, and water to form an O/W emulsion fracturing system [26,27], which is initially a uniform emulsion at 66 °C, facilitating the formation of proppant particles in deep formations. Wang [28] optimized the formula of epoxy resin emulsion and curing agent and prepared the phase transition fracturing fluid system. Additionally, nanomaterials can enhance the mechanical properties of materials [29]. Guadagno et al. [30] proposed the design of toughened self-healing supramolecular resins that achieve conductive carbon nanotubes at the electrical percolation threshold (EPT) with minimal nanofiller content. These nanotubes are dispersed within a self-healing polymer matrix to counterbalance the insulating properties of epoxy resin matrices. Zhao and Du proposed self-supporting phase transition fracturing technology based on supramolecular gel [31,32,33,34]. Based on the bionic concept of mussel, Dai et al. [35] developed a self-growing hydrogel particle system, which increases the median particle size of self-growth by 10 to 15 times, and is easy to inject and migrate far. Wang et al. [36] disclosed a deep profile control system for in situ generation of particles, which does not contain a solid phase before mixing, and can generate particles according to the fracture scale to achieve matching fracture plugging. Huang et al. [37] utilized in situ methane desorption and oxygen co-combustion to generate high-temperature, high-pressure gas shock waves. The resulting in situ shale debris particles act as self-supporting proppants, enhancing fracture conductivity under reservoir stress. These self-supporting particles exhibit mechanical properties that closely match the shale matrix, offering the natural advantage of reducing reliance on artificial proppants and simplifying operational procedures. Du et al. [38] used organic resin as raw material to prepare a temperature-sensitive self-curing resin material, which was further compounded with high-temperature-resistant fiber and other materials to construct a bridging blocking–thermally induced consolidation high-temperature plugging emulsion. The strength of the system can reach more than 6 MPa at the temperature of 90–190 °C.

Mo et al. [39] developed a novel epoxy resin self-degrading sealing agent by incorporating amine-modified additives and optimized catalysts within an epoxy–acid anhydride system. This innovative material demonstrates stable performance at 250 °C temperatures while maintaining excellent dynamic thermomechanical properties below 140 °C, proving its superior thermal resistance. At 30 MPa, the compressive crushing rate is less than 10% and the elastic deformation rate is 36.8%. Gong et al. [40] developed a microencapsulated polymer with sustained-release and viscosity-enhancing properties through reverse-phase emulsion polymerization and in situ polymerization methods. The polymer features a polyurethane prepolymer shell and a polymer core. When prepared under conditions of 800 rpm mixing speed, 50 °C reaction temperature, 4 h reaction time, and a 1:4 shell-to-core ratio, the resulting microcapsule polymer exhibits a smooth, intact shell surface with high sphericity. Zhang et al. [41] developed a novel temperature-sensitive non-reversible material (TS-LCM) particle composed of diglycidyl ether of bisphenol A (DGEBA)) and 4,4′-diaminobiphenyl, which belongs to a class of thermoresponsive shape memory polymers. TS-LCM exhibits a glass transition temperature of 70.24 °C, maintains over 99% shape fixation ratio at room temperature, and achieves 100% shape recovery above the glass transition temperature (Tg). Activated TS-LCM particles demonstrate exceptional compressive strength.

Researchers at home and abroad have carried out research on liquid–solid phase transition fracturing fluid systems, in situ self-generating particle profile control and water plugging emulsions, self-growing gel plugging emulsions, and self-consolidating resin plugging emulsions, but there is no report on self-adaptive in situ plugging technology. The drilling plugging process is different from the fracturing propping process. The proppant needs to maintain the oil and gas seepage channel in the fracture, and the proppant is required to have uniform particle size distribution and high sphericity, while the plugging process requires the plugging agent to have wide particle size distribution and excellent mechanical properties, so as to form a dense pressure-bearing plugging layer in the fracture. The self-growing hydrogel particle system can accumulate and self-grow in the reservoir. The system is easy to inject and migrate far, but the particle size distribution is uniform, the particle size is small, and there is little research on its compressive strength. Resin plugging agents are also a hot research topic at present. Aiming at the technical problem of drilling plugging in high-temperature, broken, and collapsed formations, combined with the concept of physical bridging and chemical consolidation, the self-consolidating resin plugging emulsion has a wide range of curing temperatures, but the compressive strength of the consolidated body needs to be improved, and how to discharge the formation after the formation of the consolidated body is also under-researched. Microcapsule globular polymer has a smooth shell, high degree of embedding, uniform particle size, and little description of its compressive strength. The sealing material also needs materials of different particle sizes to block the leakage. The design process of microcapsule material is complicated, and the controllable range of the particle size of sealing material is limited. In addition, the self-degrading plugging agent prepared with epoxy resin as raw material also has the limitation of particle size and is highly dependent on the scale of injected formation fractures.

Therefore, in order to solve the problem of fracture leakage in high-temperature formations and improve the one-time plugging rate, a new temperature-activated liquid–solid phase transition plugging emulsion was developed based on the principle of thermosetting resin emulsification and high-temperature crosslinking polymerization. The lost circulation system is liquid before being injected into the stratum. The lost circulation system enters into the stratum fractures with different openings and is stimulated by high temperatures. The particle strength is continuously increased during the reaction, and finally, high-strength lost circulation particles with a wide particle size distribution are generated in situ in the fractures. These particles seal the fractures in a self-adaptive matching manner and form a compact pressure-bearing sealing layer, which effectively seals the high-temperature stratum fractures and improves the success rate of plugging.

## 2. Materials and Methods

### 2.1. Materials

The reagents used in the experiments are listed in Table 1.

### 2.2. Experimental Instruments

The equipment used in the experiments is listed in Table 2.

### 2.3. Preparation of Temperature-Activated Nanomaterial Enhanced Liquid–Solid Phase Transition Plugging Emulsion

The first step was to prepare a nanomaterial-enhanced oil-in-water polymer resin emulsion, as described below.

**Figure 1:**
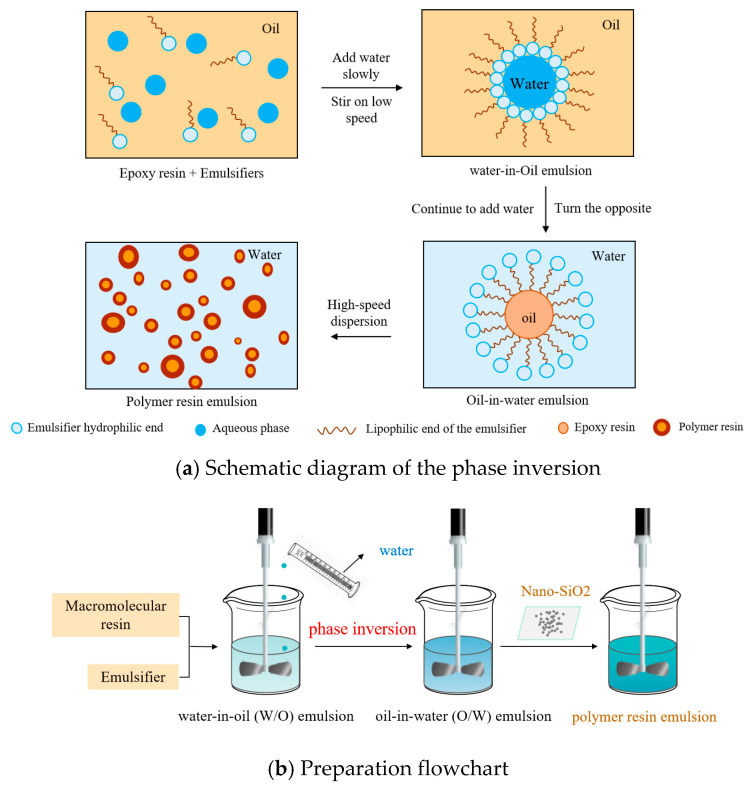
Schematic diagram of preparing oil-in-water (O/W) resin emulsion.

At room temperature, a certain amount of polymer resin XP was mixed with emulsifier CK-1, stirred uniformly, and then transferred to a water bath. The phase inversion method (Figure 1) was adopted. Under the conditions of an emulsification temperature of 40 °C and rotation speed of 600–800 r/min, distilled water was slowly added dropwise to the above mixed system. At this time, water-in-oil (W/O) emulsion was formed. Mechanical stirring was carried out for 10–30 min. When the viscosity of the system decreased suddenly, the phase inversion occurred, forming an oil-in-water (O/W) emulsion. We continued to add the remaining distilled water and nano-SiO_2_ into the system, continuing to stir mechanically for 10–20 min, and then added a certain mass of dispersant FS-1 and viscosifier ZN-1 into the emulsion in turn to prepare an oil-in-water polymer resin emulsion.

The second step was to add a crosslinker to the above emulsion, and then gradually generate high-strength nano-reinforcing particles under high-temperature conditions. For details, please refer to the following description.

**Figure 2:**
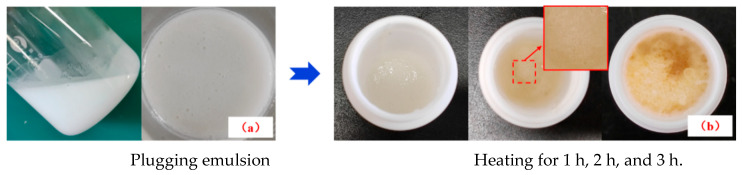

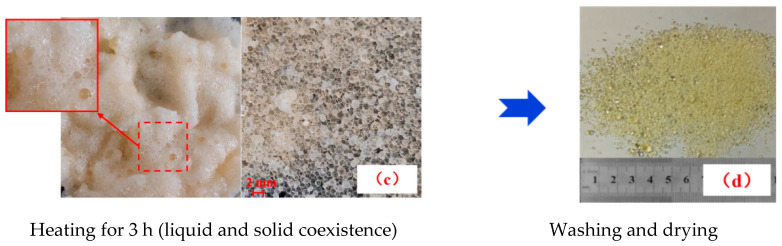
The phase transition process of temperature-activated liquid–solid phase transition plugging emulsion.

**Figure 3:**
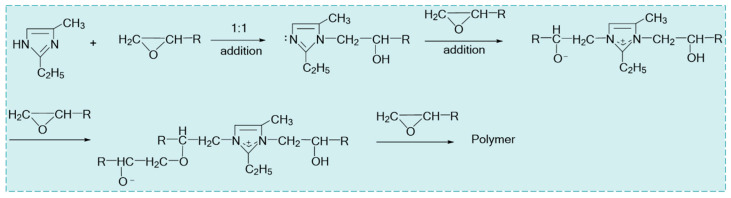
Reaction mechanism of polymer resin and crosslinker.

Under the condition that the rotating speed is 1200–1500 r/min, a proper amount of crosslinker JL-1 is added into the oil-in-water resin emulsion system, and the system is stirred for 30 to 50 min to form a temperature-activated liquid–solid phase transition plugging emulsion (Figure 2a), with the reaction mechanism of the polymer resin and the crosslinker shown in Figure 3. The plugging emulsion is transferred to a reaction kettle and reacted in an oven at a constant temperature for 2–3 h to obtain a mixed system of a non-phase transition system and solid particles (Figure 2c). After washing and drying, the product in situ self-generated plugging particles are obtained (Figure 2d).

### 2.4. Factors Influencing of Particle Formation

In order to adapt to fractures with different apertures in the formation, the in situ self-generated plugging particles prepared in the experiment are required to have a wide particle size distribution to achieve a good plugging effect. The influences of different factors such as the content of emulsifier, the content of dispersant, the content of crosslinking agent, the content of distilled water, and the content of thickener on the particle size distribution were explored. The in situ self-generated particles were screened, and the particle size distribution curve was plotted. The characteristic value D(90) of the particle size distribution was calculated to obtain the optimal formulation. Then, the influencing factors of phase transition temperature, phase transition time, and compressive strength on the regulation law of particle forming were investigated, and finally, an optimal formulation was formed.

### 2.5. Structural Characterization Test

The thermal stability of in situ plugging agent was measured by Fourier transform infrared spectroscopy (FTIR) with the scanning range of 550–4000 cm^−1^. The thermal stability of the in situ plugging agent was measured by thermogravimetry–differential thermal analysis at a temperature of 30–800 °C and a heating rate of 10 °C/min. The gas atmosphere used in the TGA analysis of the paper was nitrogen. The microstructure of the in situ plugging particles was observed by scanning electron microscopy (SEM) at an accelerating voltage of 10 kV. The samples were subjected to gold sputtering treatment before the SEM analysis. The coalescence process of oil droplets in the oil-in-water polymer emulsion and the crosslinking and curing process of the plugging emulsion were observed by optical microscope.

### 2.6. Performance Test

#### 2.6.1. Compressive Strength Test

The compressive strength of in situ plugging particles before and after aging at 120 °C/16 h was tested by universal pressure testing machine, and the crushing test was carried out under the pressure of 60 MPa. The D(90) particle size degradation rate (*SC*) was used as the evaluation index to evaluate the compressive strength of plugging materials. The smaller the *SC* value was, the higher the compressive strength was. Before the crushing experiment, the D(90) before was calculated by sieving the in situ self-generated particles, and then the sample was placed into the sample device of the universal testing machine. After the pressure was stabilized for 5 min at 60 MPa, the universal testing machine was closed, and the D′(90) after the experiment was measured.

(1) $$SC=\frac{\begin{array}{c} D(90) \end{array}-D^{\prime }(90)}{D(90)}\times 100\%$$

In the Formula (1), *SC* is the particle size degradation rate, %; D(90) is the characteristic parameter of particle size before compression, mm; and D′(90) is the characteristic parameter of particle size after compression, mm.

#### 2.6.2. Temperature Resistance Test

Due to the high-temperature environment of the formation, higher requirements are put forward for the use temperature of the in situ autogenous plugging agent. In this paper, the temperature resistance of the in situ autogenous plugging agent was evaluated by testing its strength and morphology changes after high-temperature aging. The specific test method is as follows: Put the in situ autogenic particles into the drilling fluid, age them at 120 °C, 140 °C, 160 °C, and 180 °C for 16 h, take out the aging samples, rinse them, dry them, and weigh them. Calculate the quality loss of the self-generated plugging particles before and after aging (as shown in Equation (2)), observe the morphological changes in the plugging material before and after aging, and judge the temperature resistance of the plugging agent by combining the two.

(2) $$Mass\;loss=\frac{\begin{array}{c} m_{Pre-aging} \end{array}}{m_{Post-aging}}\times 100\%$$

#### 2.6.3. Rheological Properties Test

The rheological properties of the temperature-activated liquid–solid phase transition sealing system were evaluated by calculating the apparent viscosity (*AV*), plastic viscosity (*PV*), and yield point (*YP*). The injected fluid must maintain adequate fluidity while possessing sufficient viscosity to ensure effective penetration into the formation, thereby minimizing fluid loss and establishing a stable, efficient seal.

At room temperature, the sealing system was placed in a reaction vessel and tested using a six-speed rotational viscometer. The dial reading (*θ*_1_) was recorded after stabilization at 600 rpm, followed by another reading (*θ*_2_) at 300 rpm. The apparent viscosity (*AV*), plastic viscosity (*PV*), and yield point (*τ*) were then calculated according to Equations (3), (4) and (5), respectively.

The apparent viscosity (*AV*) is calculated using the following equation:

(3) $$AV=\frac{\begin{array}{c} 1 \end{array}}{2}\times \theta_{1}$$

The plastic viscosity (*PV*) is calculated using the following equation:

(4) $$PV=\theta_{1}-\theta_{2}$$

The yield point (*τ*) is calculated using the following equation:

(5) τ=AV−PV

#### 2.6.4. Compatibility of Drilling Fluid Test

Compatibility is an important indicator for evaluating plugging materials. Therefore, the compatibility of the temperature-activated liquid–solid phase transition plugging emulsion was evaluated by adding different contents of drilling fluid base slurry. The specific implementation method is as follows: 5%, 10%, and 15% of the drilling fluid base slurry were, respectively, added into the temperature-activated liquid–solid phase transition plugging emulsion. After stirring evenly, the mixture was put into a rolling aging furnace, and the temperature required for the reaction was set; then, we observed whether particles formed. The formulation of the drilling fluid base slurry used is as follows: 20 L of water + 40 g of sodium carbonate + 800 g of bentonite.

#### 2.6.5. Fracture Plugging Performance Test

The fracture plugging simulation experiment is an important means to evaluate the plugging effect of plugging materials. The plugging experiment research on the temperature-activated liquid–solid phase transition plugging emulsion was carried out by using a high-temperature and high-pressure long-fracture-plugging simulation experimental device to explore the fracture-plugging effect of the in situ self-generated plugging particles. The same set of temperature-activated liquid–solid phase transition plugging emulsion was adopted to plug the wedge-shaped fracture modules with different apertures of 1 mm, 3 mm, and 5 mm, respectively. After the device was assembled, it was heated to the phase transition temperature, and the temperature-activated liquid–solid phase transition plugging emulsion was pumped into the fractures from the drilling fluid kettle body. After standing for a period of time, due to the high-temperature stimulation, the liquid-phase plugging emulsion self-generated plugging particles in situ in the fractures. Then, the temperature-activated liquid–solid phase transition plugging emulsion was replaced with the drilling fluid system, and the pressure was continuously increased to test the pressure-bearing capacity of the plugging until the plugging was broken through. The obtained maximum pressure is the pressure-bearing capacity of the plugging.

## 3. Results and Discussion

### 3.1. Study on the Forming Regulation Factors of the Temperature-Activated Liquid–Solid Phase Transition Plugging Emulsion

Based on the experimental method as described in Section 2.4, the experiment explored the influencing factors such as phase transition temperature, phase transition time, particle size distribution, and compressive strength on the regulation law of particle forming and, finally, formed an optimal formulation.

#### 3.1.1. Phase Transition Temperature Analysis

**Figure 4:**
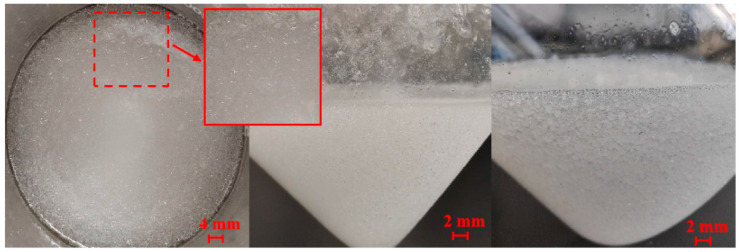
Phase transition process of medium-temperature crosslinker.

The effect of curing system type on the phase transition temperature of the temperature-activated liquid–solid phase transition plugging emulsion was investigated. Plugging particles were prepared using different types of crosslinking agents. It was found that the phase transition temperature range of the plugging particles prepared using the medium-temperature crosslinking agent was 50–90 °C, while that of the particles prepared using the high-temperature crosslinking agent was 80–120 °C (as shown in Figure 4). The results show that the phase transition temperature range is directly proportional to the applicable temperature of the selected crosslinking agent.

#### 3.1.2. Phase Transition Time Analysis

The temperature-activated liquid–solid phase transition sealing system undergoes phase transformation under high-temperature stimulation until the particle size distribution and compressive strength stabilize, with this duration defined as the phase transition time. This study investigates the influence of phase transition temperature on the phase transition time of the system (as shown in Table 3). All experimental groups utilized the same crosslinker to exclusively examine the effect of phase transition temperature on the transformation kinetics.

**Figure 5:**
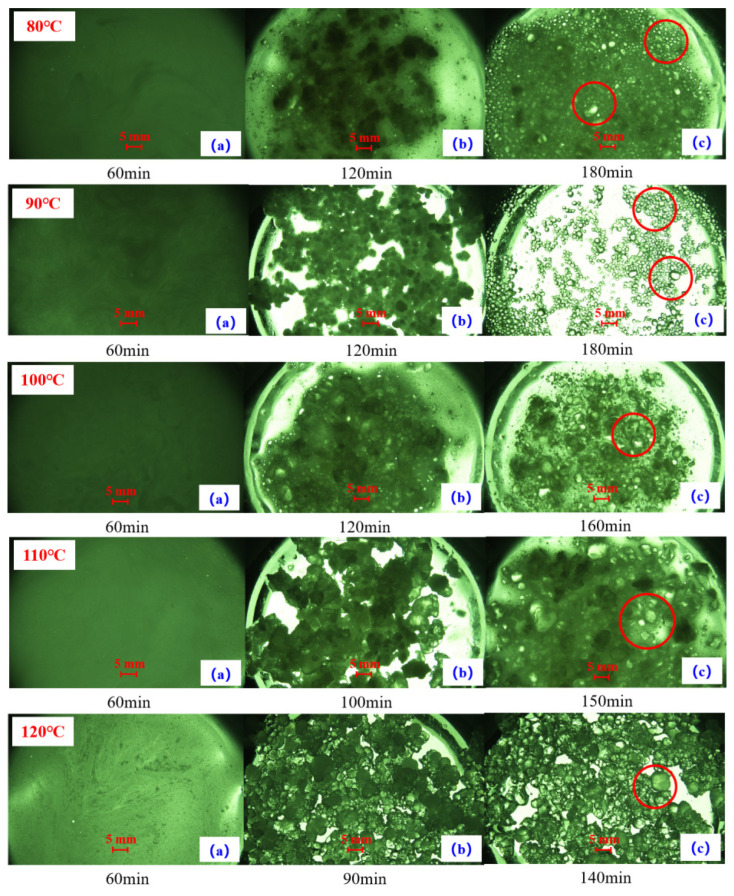
The phase transition time of the plugging emulsion at varying temperatures.

As shown in Figure 5, phase transition times corresponding to different temperatures were systematically recorded, revealing three distinct transition stages: the crosslinking and coalescence stage (a), initial solidification stage (b), and complete curing stage (c). During phase (a) at different phase transition temperatures, the system remains liquid with a transition time of 60 min. In phases (b) and (c), distinct transition times are observed corresponding to varying temperatures. Elevated temperatures accelerate molecular thermal motion, resulting in faster curing rates and, consequently, reduced phase transition durations. The red circle in Figure 5 highlights phase (c), where high-strength plugging particles form. Notably, particle size distribution varies with transition temperature: lower temperatures yield non-agglomerated particles with wider size distribution, while 120 °C produces larger but partially agglomerated particles. Higher transition temperatures shorten particle formation time. The temperature-activated liquid–solid transition system demonstrates optimal performance within 80–120 °C, achieving complete particle formation within 2–3 h.

The results demonstrate that within the 80–90 °C temperature range during stage (a), the system exists as an interconnected liquid network. Complete phase transition occurs after 180 min (Figure 5c), yielding high-strength in situ self-generating plugging particles in a transparent granular state. Notably, the phase transition duration progressively decreases with increasing temperature: at 100 °C, 110 °C, and 120 °C, the complete solidification times reduce to 160, 150, and 140 min, respectively. Throughout this temperature spectrum (80–120 °C), the system consistently produces effective in situ self-generating plugging particles capable of fracture sealing. The temperature-activated liquid–solid phase transition plugging emulsion achieves complete transformation within an adjustable timeframe of 140–180 min, demonstrating its robust temperature-responsive performance.

#### 3.1.3. Particle Size Distribution Analysis

In view of the multi-scale characteristics of the formation fractures, the influence laws of factors such as the content of emulsifier, the content of dispersant, the content of crosslinking agent, the content of distilled water, and the phase transition temperature on the particle size distribution of the self-generated plugging particles were analyzed, so that the particle size of the self-generated plugging particles in the fluid loss formation is relatively large and the distribution is relatively wide (0.1–5 mm), which is beneficial to the formation of a dense pressure-bearing plugging layer. The D(90) rule is to ensure that the D(90) value of the plugging material is slightly smaller than or equal to the average aperture of the fluid loss channel. Taking the D(90) rule as an important basis for the particle size of the plugging material, and then combining with other influencing factors, the optimal formulation of the self-generated plugging particles was comprehensively determined through overall consideration.

1. Content of the emulsifier

**Figure 6:**
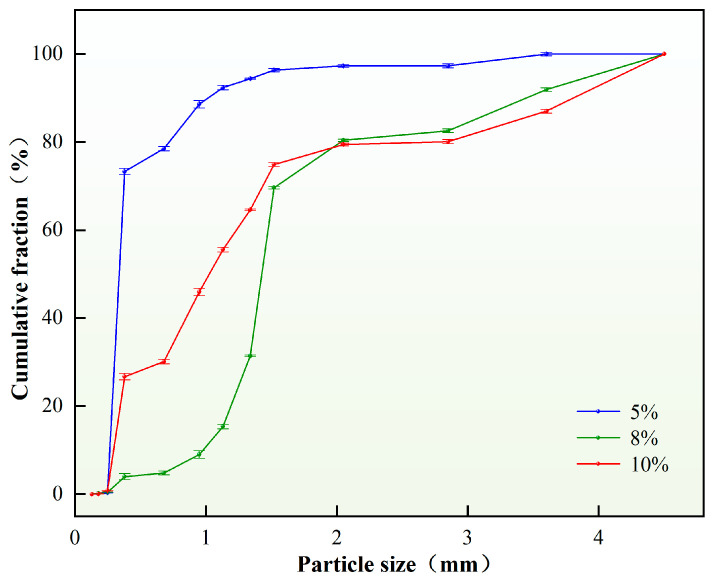
Particle size distribution of plugging particles under different emulsifier content.

From a thermodynamic perspective, emulsions are inherently unstable systems characterized by high interfacial energy, wherein droplets tend to spontaneously coalesce to reduce interfacial energy, driving the system toward thermodynamic equilibrium. However, uncontrolled coalescence ultimately leads to emulsion breakdown. Therefore, the incorporation of appropriate emulsifier content serves to reduce interfacial tension, thereby enhancing thermodynamic stability while maintaining kinetic stability, consequently improving overall emulsion stability. The study systematically investigated the influence of emulsifier content on the particle size distribution of self-generated plugging particles. Under otherwise constant conditions, variations in emulsifier content were examined, with results presented in Figure 6.

As evidenced by Figure 6, when the emulsifier content ranges between 5 and 10 wt.%, the in situ-generated plugging particles form discrete solid particulates without significant agglomeration. The D(90) value of solid particles exhibits a positive correlation with increasing emulsifier content, primarily attributable to enhanced oil–water interfacial film strength at optimal concentrations, which stabilizes droplets and increases average particle size. However, excessive emulsifier content (>10 wt.%) leads to surplus molecules participating in phase inversion as micelles rather than interfacial stabilization, consequently reducing emulsion stability and inducing severe particle agglomeration. Based on comprehensive analysis, an emulsifier content of 8 wt.% was selected as the optimal concentration, effectively satisfying fracture sealing requirements. Particle size distribution analysis yielded a D(90) value of 3.40 mm.

2. Content of the dispersant

**Figure 7:**
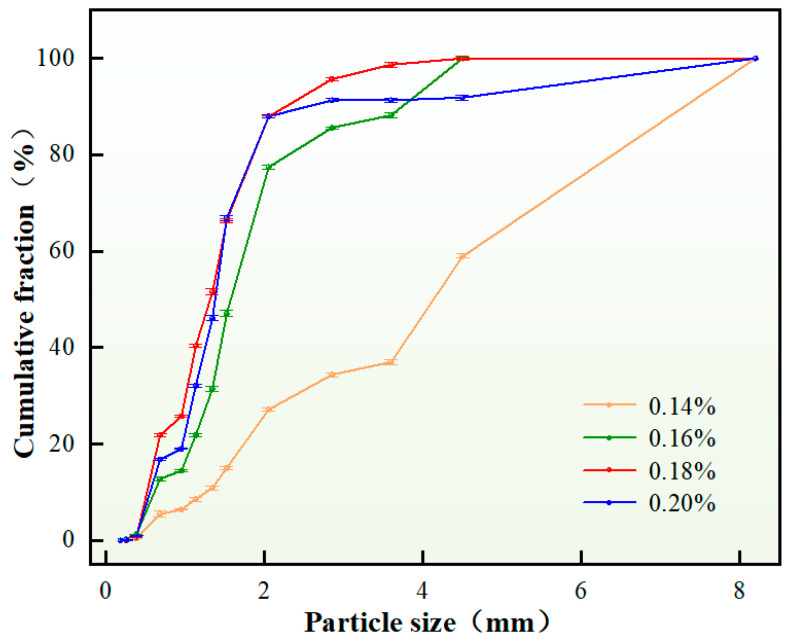
Particle size distribution of plugging particles under different dispersant content.

Dispersants, as polymeric molecules, function to reduce droplet sedimentation and coalescence within the system, delay particle agglomeration, and maintain prolonged dispersion stability, thereby enhancing overall system stability. The experimental investigation examined the influence of dispersant content on the particle size distribution of self-generated plugging particles. Under otherwise constant conditions, variations in dispersant content were systematically evaluated, with the results presented in Figure 7.

As demonstrated in Figure 7, when the dispersant content was 0.14 wt.%, insufficient dispersion resulted in molecular aggregation of droplets due to inadequate particle separation, leading to significant agglomeration with only minimal formation of discrete plugging particles. Increasing the dispersant content to 0.16–0.20 wt.% promoted stable solid particle generation, with both average particle size and D(90) values exhibiting a decreasing trend. This phenomenon is attributed to enhanced droplet stabilization and reduced interfacial tension at optimal dispersant concentrations, which prevented coalescence into larger particles and ensured uniform dispersion. Based on comprehensive analysis, a dispersant content of 0.16 wt.% was selected as the optimal concentration, effectively satisfying fracture sealing requirements. Particle size distribution analysis yielded a D(90) value of 3.71 mm.

3. Content of the crosslinker

**Figure 8:**
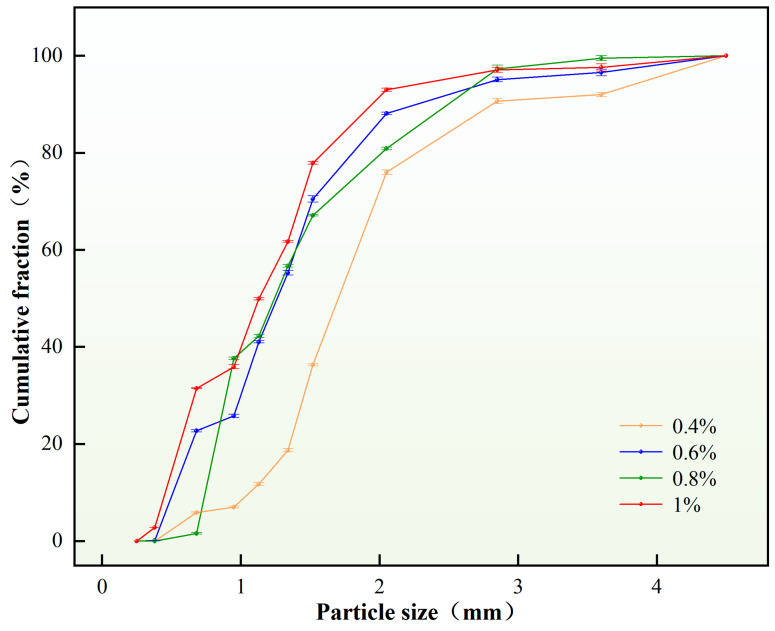
Particle size distribution of plugging particles under different crosslinker contents.

The crosslinker primarily functions to initiate crosslinking and curing reactions with polymer resins, forming an insoluble three-dimensional network structure. Insufficient crosslinker content prevents complete reaction of the polymer resin, failing to establish the desired crosslinked polymer network. Conversely, excessive crosslinker content results in unreacted surplus molecules that compromise the structural integrity of the three-dimensional network. This study systematically investigated the influence of crosslinker content on the particle size distribution of self-generated plugging particles. Under otherwise constant experimental conditions, variations in crosslinker content were examined, with the results presented in Figure 8.

The experimental results in Figure 8 demonstrate that within the crosslinking agent concentration range of 0.4–1.0 wt.%, well-dispersed particles formed without agglomeration. In this optimal range, the active hydrogen groups of the crosslinking agent completely reacted with epoxy groups, resulting in complete crosslinking that formed high-strength three-dimensional network macromolecules. Both the D(90) value and average particle size exhibited a consistent decreasing trend with increasing crosslinking agent concentration. Based on comprehensive analysis, a crosslinking agent content of 0.4 wt.% was determined to be the optimal formulation, effectively satisfying the fracture sealing requirements. Particle size distribution analysis confirmed a D(90) value of 2.80 mm for the resulting particles.

4. Content of the distilled water

**Figure 9:**
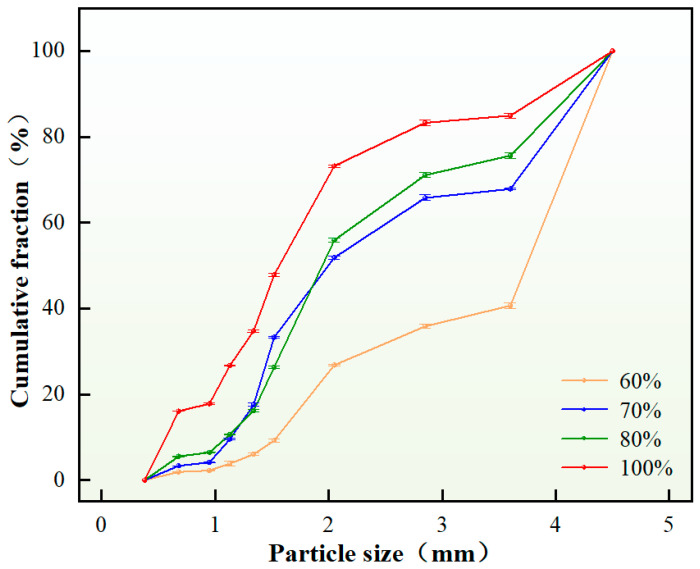
Particle size distribution of plugging particles under different distilled water content.

The experiment explored the influence of the content of distilled water on the particle size distribution of the self-generated plugging particles. On the premise that other factors and conditions remained unchanged, the content of distilled water was varied, and the results are shown in Figure 9.

As shown in Figure 9, when the distilled water content was 60%, the self-generated plugging particles formed partially agglomerated particles with relatively large average size. This phenomenon primarily resulted from insufficient water content, which deteriorated emulsion dispersibility and promoted droplet coalescence through enhanced molecular collisions, ultimately leading to particle agglomeration. In contrast, within the 70–100% water content range, increasing water volume facilitated complete reactions among water, emulsifier and polymer resin, enabling effective phase inversion from water-in-oil (W/O) to oil-in-water (O/W) emulsion. The stabilized emulsion system generated solid particles with controlled size distribution, exhibiting decreased average droplet diameter and reduced D(90) values with increasing water content. In conclusion, choosing the content of distilled water to be 70% as the appropriate content of distilled water can better meet the crack criteria. Through the analysis and calculation of the particle size distribution diagram, the D(90) value obtained is 3.96 mm.

5. Content of the viscosifier

**Figure 10:**
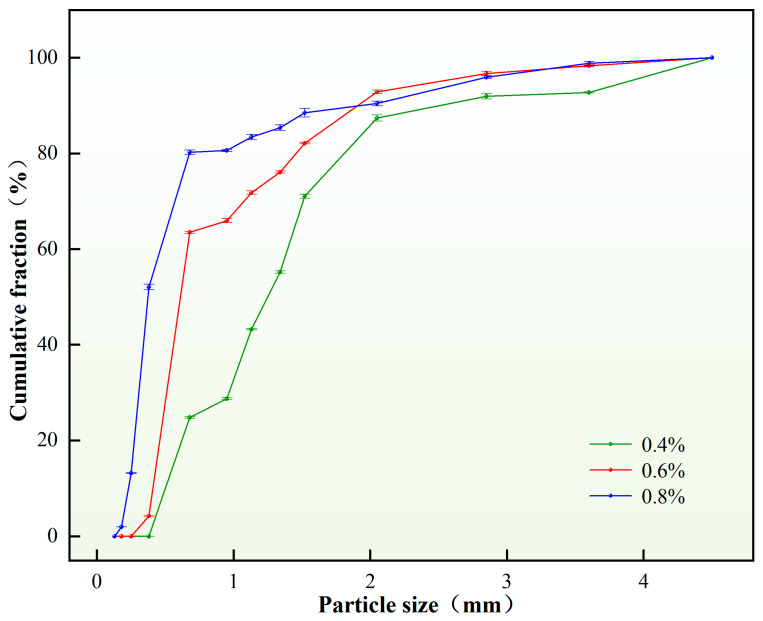
Particle size distribution of plugging particles under different viscosifier content.

The experiment investigated the influence of viscosifier content on the particle size distribution of self-generated plugging particles. Under constant other parameters, variations in viscosifier content were tested, with the results illustrated in Figure 10.

As evidenced by Figure 10, when the viscosifier content was 0.2 wt.%, particle formation occurred alongside agglomeration phenomena, indicating an extremely unstable emulsion system. An insufficient viscosifier content (0.2 wt.%) compromised the emulsion stability, causing droplet coalescence or sedimentation during the crosslinking and curing process of the plugging emulsion, thereby increasing particle size. Within the optimal viscosifier content range of 0.4–0.8 wt.%, the plugging emulsion maintained relative stability, forming discrete solid particles without agglomeration. Notably, the D(90) value exhibited a decreasing trend with increasing viscosifier concentration in this optimal range. In summary, a viscosifier content of 0.4 wt.% was selected as the optimal concentration, demonstrating satisfactory compliance with fracture sealing requirements. Through particle size distribution analysis, the corresponding D(90) value was determined to be 2.35 mm.

#### 3.1.4. Compressive Strength Analysis

1. The influence of nano-silica on compressive strength

Samples without nano-silica and those with nano-silica were, respectively, selected to prepare plugging particles. The plugging particles were aged at 120 °C, 140 °C, 160 °C, and 180 °C for 16 h, followed by crushing tests under a pressure of 60 MPa. The experimental results are shown in Table 4. The results show that the particle size degradation rate of the particles without the addition of nano-silica was 5.25–10.84%, and the compressive strength of the particles with the addition of nano-silica was significantly improved, with a particle size degradation rate of 3.07–5.41%. Compared with traditional plugging materials, these plugging particles exhibit good mechanical properties and excellent temperature resistance. They exhibit almost no breakage under high-pressure differential conditions of 60 MPa, remaining virtually unchanged before and after compression. Nano-silica (SiO_2_) has an extremely high specific surface area, which generates a very strong interfacial bonding force between nanoparticles and the resin. Additionally, it can fill the voids and defects between the molecular chains of the matrix material, reducing the internal stress concentration points. When microcracks propagate into nanoparticles, their propagation paths will deflect or detour, thereby prolonging the crack propagation path and consuming more energy. Therefore, the mechanical properties of the particles should be improved.

2. Phase transition time

**Figure 11:**
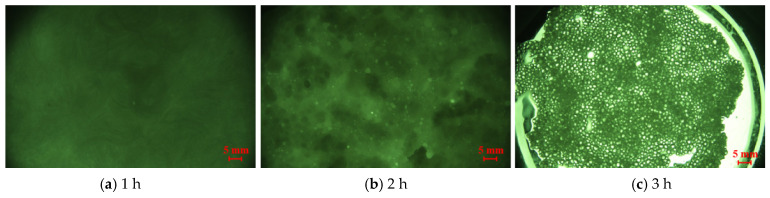
Plugging particles formed under different phase transition times.

The plugging particles synthesized at different phase transition times were shown in Figure 11, with phase transition times recorded starting from 1 to 3 h. No particle formation was observed after maintaining a constant temperature for 1 h. Upon extending the duration to 2 h, droplet-like oil beads with enhanced strength became visible, accompanied by the generation of small, milky-white particles exhibiting low hardness. After 3 h of constant temperature, a multiphase hybrid system composed of liquid-phase components and spherical solid particles was formed. The compressive strength of particles increases with the extension of time.

#### 3.1.5. Optimal Formulation of the Temperature-Activated Liquid–Solid Phase Transition Plugging Emulsion

By systematically investigating the effects of emulsifier content, dispersant content, crosslinker content, distilled water content, viscosifier content, and phase transition temperature on the particle size distribution of the self-generated plugging particles, the optimal formulation for the temperature-activated liquid–solid phase transition plugging emulsion was determined as follows: emulsifier content of 8 wt.%, dispersant content of 0.16 wt.%, crosslinker content of 0.4 wt.%, distilled water content of 70 wt.%, viscosifier content of 0.4 wt.%, and nano-silica content of 2.0 wt.%. Here, the percentage contents of all components refer to their proportions relative to the total polymer resin content.

**Figure 12:**
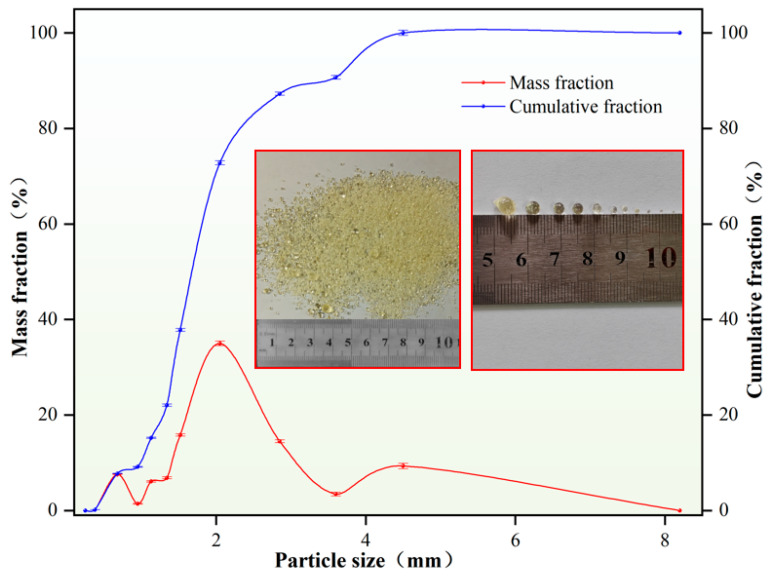
Self-generated plugging particles.

Self-generated plugging particles exhibited a compressive strength 60 MPa, with primary particle sizes distributed between 0.1 and 5 mm. The particle size distribution was concentrated in the 8–20 mesh range, accounting for 65.06% of the total. The particle size distribution curve yielded D10 = 1.00 mm, D50 = 1.38 mm, and D(90) = 3.51 mm, indicating suitability for bridging, filling, and sealing fractures in leakage zones with varying apertures, as demonstrated in Table 5 and Figure 12.

**Figure 13:**
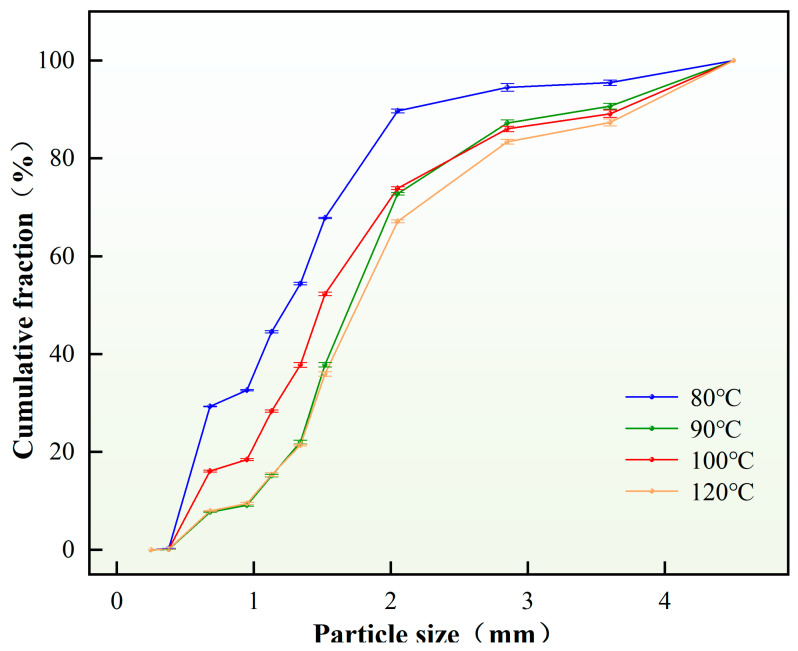
Particle size distribution of plugging agents under different temperatures.

The formation temperature applicable to the liquid–solid phase transition plugging emulsion was explored. Experimental results are presented in Table 6 and Figure 13. At low temperatures (<80 °C), the system exhibits elevated viscosity with incomplete phase inversion of the emulsion, leading to agglomeration risks. With increasing temperature, the viscosity decreases, enabling complete emulsification reaction. During subsequent crosslinking and curing, the phase transition fluid undergoes progressive coalescence. Through Brownian motion, droplets collide and merge, transforming multiple micro-droplets into macro-droplets with enhanced structural integrity. Finally, in situ self-generated plugging particles with high strength were formed.

As can be seen from Table 6 and Figure 13, solid particles with good dispersion effect can be formed within the temperature range of 80 to 120 °C, and there is no adhesion phenomenon. The main difference lies in the size of the particle diameter. The D(90) value increases with the rise in the phase transition temperature. The main reason for this is that the increase in temperature accelerates the coalescence between droplets and also speeds up the process of the crosslinking and curing reaction. Therefore, the particle diameter generally shows an increasing trend. However, when the temperature is too high (>120 °C), the probability of droplet aggregation in the system increases, the number of large droplets gradually increases, and the emulsion system is damaged, leading to caking. In summary, within the formation temperature range of 80–120 °C, the system satisfactorily meets fracture sealing requirements. The self-generated plugging particles achieve a controlled size distribution of 0.1–5 mm, enabling effective bridging, filling, and sealing in fractures with varying apertures.

### 3.2. Infrared Spectroscopy Analysis

**Figure 14:**
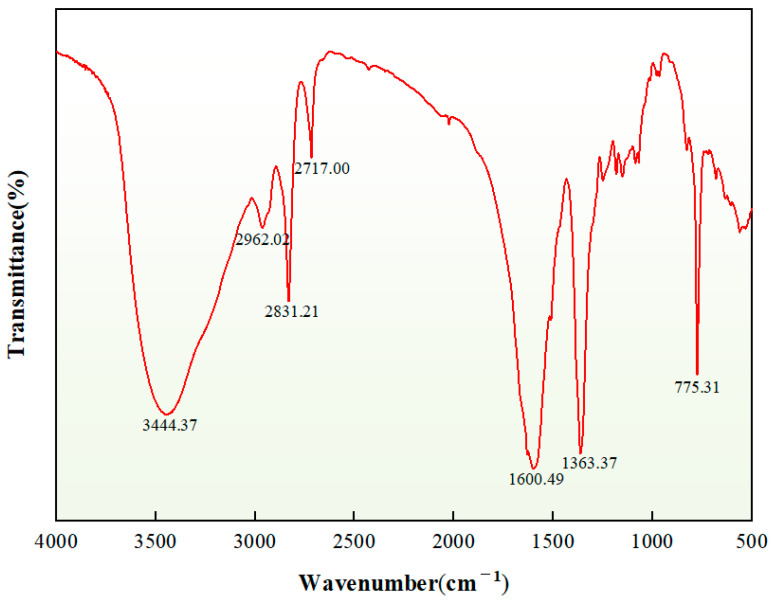
FTIR spectrum of self-generated plugging particles.

After washing and drying the plugging particles, the product was tested with a Bruker Tensor 27 infrared spectrometer, as shown in Figure 14. The C-N stretching vibration appears at 1363 cm^−1^, while the modified cycloaliphatic amine exhibits a characteristic C-N peak at 1375 cm^−1^. The C-N stretching vibration peak in the product shifts to a lower wavenumber by 12 cm^−1^, which originates from the formation of more stable C-N-C bonds after the ring-opening reaction between amino groups and epoxy groups. This shift is caused by the electron cloud density deviation, which leads to a decrease in the bond force constant; the C-H stretching vibration of saturated hydrocarbon at 2717 cm^−1^, 2831 cm^−1^, and 2962 cm^−1^; the C = C stretching vibration of heterocycle at 1600 cm^−1^; and the O-H stretching vibration at 3444 cm^−1^. A new O-H stretching vibration peak appears in the product. Intramolecular hydrogen bonding weakens the bond force constant, which also causes a red shift of this peak. The N atom in the crosslinker has an addition reaction with the epoxy group to finally form an O-H bond. With the consumption of the epoxy group, the characteristic peak of the epoxy group at 910 cm^−1^ disappears, which indicates that the epoxy group completely reacted with the crosslinker and had a ring-opening polymerization reaction with the crosslinker to form a three-dimensional network structure, and the raw material was transformed into high-strength in situ self-generated plugging particles.

### 3.3. Thermogravimetric Analysis

**Figure 15:**
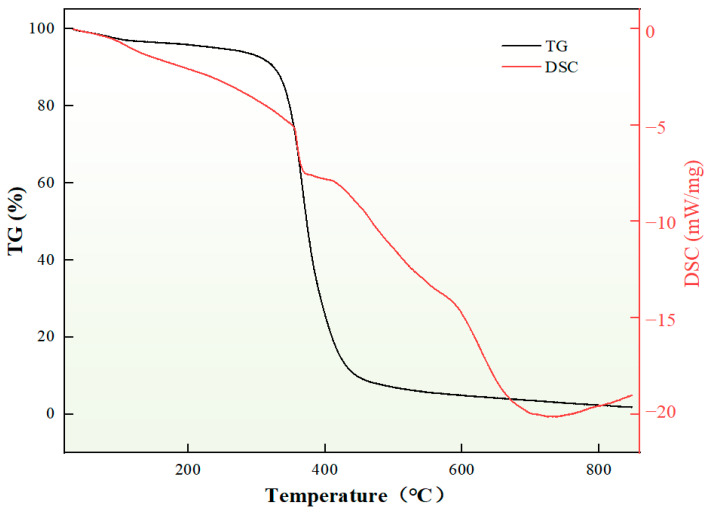
Thermogravimetric curve analysis of self-generated plugging particles.

The thermogravimetric analyzer was used to determine the mass change in the self-generated plugging agent with temperature. Figure 15 shows the thermogravimetric curve analysis of the self-generated plugging particles. The thermal degradation process can be divided into three distinct stages. The first stage, occurring below 300 °C, shows a minimal weight loss of 0.8%, which can be attributed to the evaporation of adsorbed moisture and minor organic volatiles from the sample material. The second stage, between 300 and 450 °C, represents the primary decomposition phase of the sample material. In the third stage (450–850 °C), the residual mass stabilizes at 2.02%, indicating completion of the thermal reaction process. These results demonstrate that the in situ self-generating plugging agent exhibits excellent thermal stability, maintaining its structural integrity without significant thermal degradation at temperatures below 300 °C. This thermal property ensures reliable performance in high-temperature applications.

### 3.4. Optical Microscope Analysis

**Figure 16:**
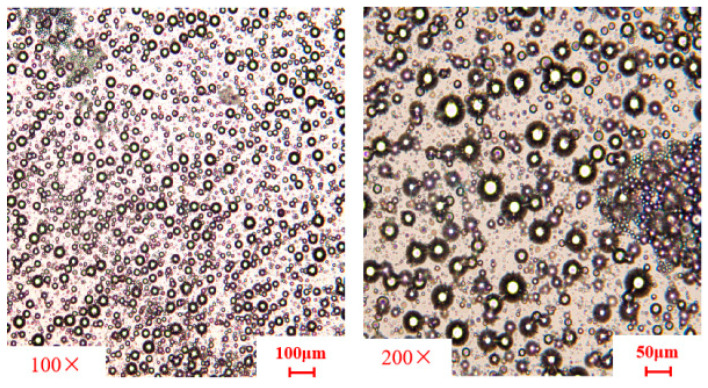
Micrographs of oil-in-water polymer resin emulsion.

Figure 16 presents optical micrographs of the oil-in-water polymer resin emulsion at room temperature. The system maintains uniform dispersion, exhibiting a mixed-phase composition of phase transition fluids and non-phase transition fluids. Notably, the immiscibility between the two liquid phases is clearly observable, with discrete droplets of varying sizes present. This phase separation behavior confirms that the phase transition fluid can form incompatible solid materials within the non-phase transition fluid, thereby achieving effective fracture sealing in geological formations.

**Figure 17:**
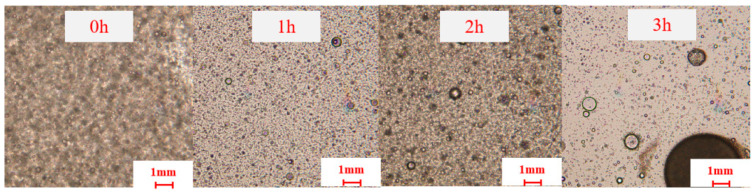
Crosslinking and curing process of plugging emulsion.

As shown in Figure 17, upon further heating to 90 °C, the plugging emulsion undergoes crosslinking and curing reactions under elevated temperature conditions. Microscopic observation clearly reveals that the non-phase transition fluid remains in liquid phase, forming flow channels, while the phase transition fluid progressively transforms from the liquid to the solid phase.

### 3.5. Scanning Electron Microscope Analysis

**Figure 18:**
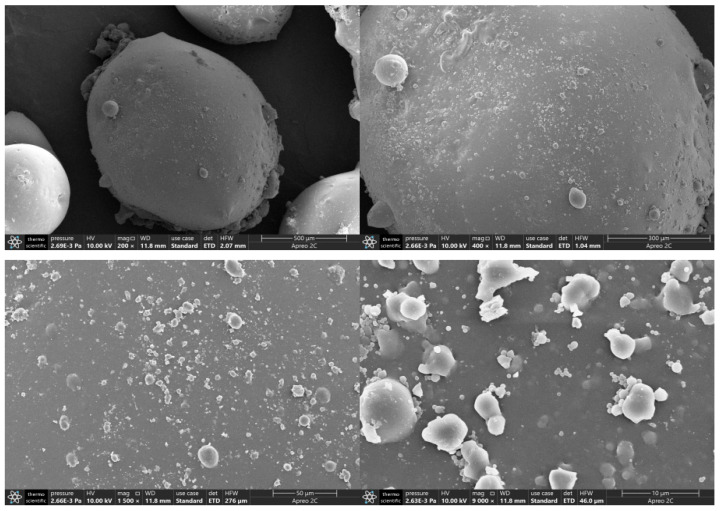
SEM images of self-generated particles.

The self-generated plugging particles were washed, dried, and shot after metal spraying treatment. Under the condition of an accelerating voltage of 10 kV, images were magnified by 200×, 400×, 1500×, and 9000× using a scanning electron microscope, as shown in Figure 18. It can be seen from Figure 18 that there are no obvious depressions and fractures on the surface of the in situ self-generated plugging particles, indicating that a compact network structure is formed inside after the crosslinking reaction, and the external solid particles are generally spherical rather than linear and massive. It can be observed that the surface of solid particles is not completely smooth, so there is a certain friction between particles. This might be caused by nano-silica adhering to the surface. When plugging formation fractures, the in situ self-generated plugging material is not easily to taken away by the fluid, which can achieve effective plugging.

### 3.6. Temperature Resistance Analysis

**Figure 19:**
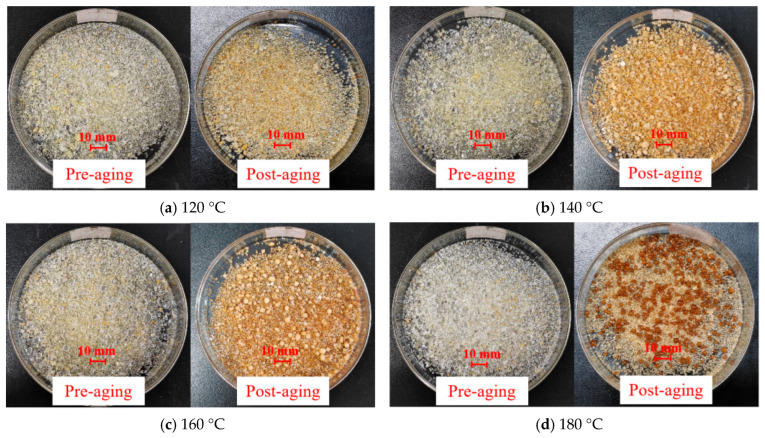
Morphology change in particles before and after aging.

**Figure 20:**
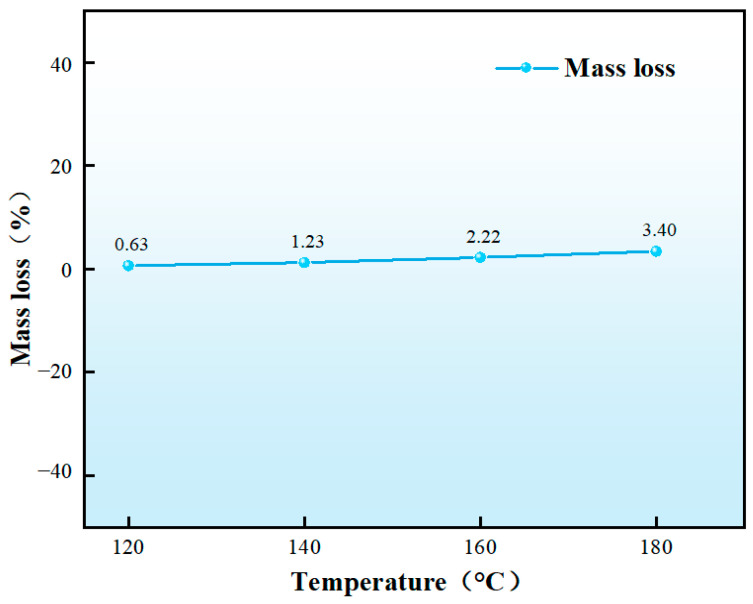
Mass loss curve of the particles after aging.

Figure 19 demonstrate significant color darkening after high-temperature aging, with the particles turning yellowish, indicating further curing of the solid particles during aging. Higher temperatures resulted in more pronounced aging effects, as evidenced by deeper coloration. As shown in Figure 20, after washing and drying, the mass loss of in situ self-generating particles was calculated, showing values of 0.63%, 1.23%, 2.22%, and 3.40% after aging at 120 °C, 140 °C, 160 °C, and 180 °C, respectively. The mass loss remained relatively low overall. Combined with Table 5, the degradation rate is 5.41% after aging at 180 °C. These findings confirm that the particles exhibit excellent thermal resistance, maintaining structural integrity up to 180 °C while demonstrating effective compressive performance at 60 MPa, making them highly suitable for sealing fractures in high-temperature formations.

### 3.7. Rheological Properties Analysis

At room temperature, rheological characterization of the identical temperature-activated liquid–solid phase transition plugging emulsion was conducted through viscosity measurements. Two experimental replicates were performed, with parameters calculated according to Equations (3)–(5) in Section 2.6.3 (Table 7). The system demonstrated favorable flow characteristics with apparent viscosities of 83.5 mPa·s and 84.0 mPa·s, and a plastic viscosity of 43.0 mPa·s, indicating excellent mobility for penetration into fracture channels. It also ensures excellent pumping performance.

### 3.8. Compatibility of Drilling Fluids Analysis

**Figure 21:**
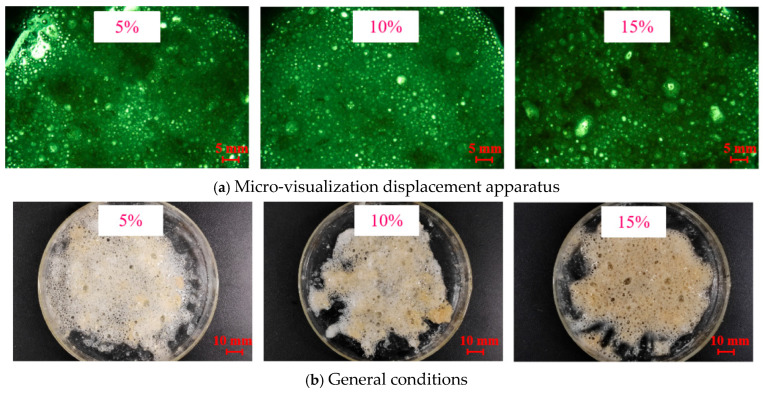
Solidification reaction of plugging emulsion with different content of drilling fluid.

Drilling fluid base slurries with contents of 5%, 10%, and 15% were, respectively, added to the plugging emulsion. After uniform stirring, the mixtures were placed in a rolling aging oven, and the aging temperature was 90 °C. The micro-visualization device was used to record the formation of particles after adding them to the drilling fluid system (Figure 21a). The results show that self-generated plugging particles can be formed in the plugging emulsions with 5%, 10%, and 15% drilling fluid base slurries, indicating good compatibility between the plugging emulsion and the drilling fluid, which can be used for formation plugging.

### 3.9. Fracture Plugging Performance Analysis

**Figure 22:**
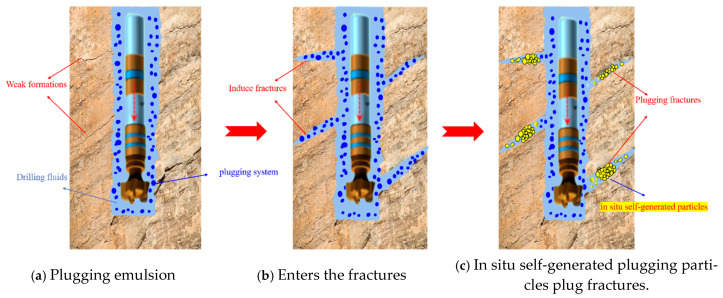
Schematic diagram of the plugging process of the plugging emulsion.

As shown in Figure 22, the temperature-activated liquid–solid phase transition plugging emulsion is composed of phase transition fluid and non-phase transition fluid, where the phase transition fluid is droplets formed by a polymer resin emulsion and crosslinking system, and the non-phase transition fluid is water. Stimulated by the in situ high temperature in the loss zone, this liquid system can adaptively enter fractures with different openings without requiring clear knowledge of the fracture opening in advance. The phase transition droplets continuously coalesce and undergo crosslinking and solidification reactions, with their particle size and strength increasing continuously. When the strength reaches a level that prevents further coalescence of particles, the particle strength continues to increase while the particle size remains unchanged, and the liquid phase transforms into a solid phase, eventually in situ generating high-strength plugging particles with different particle sizes in the fractures to intelligently match the fracture dimensions. The non-phase transition fluid in the fractures flows into the deep formation under pressure difference, while the non-phase transition fluid in the wellbore still occupies the original space.

**Figure 23:**
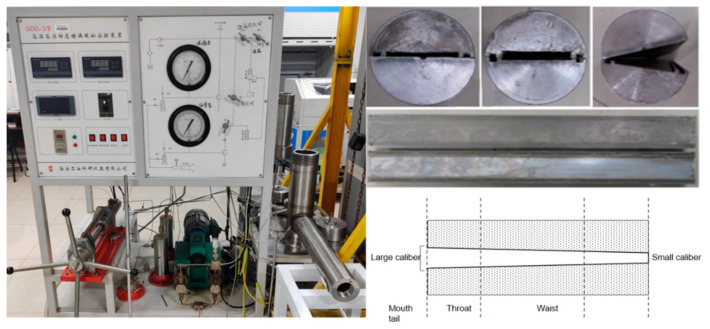
High-temperature and high-pressure fracture plugging simulation experimental device.

The high-temperature and high-pressure fracture plugging simulation experimental device was used to simulate fracture plugging (Figure 23). With the same temperature-activated liquid–solid phase transition plugging emulsion, wedge-shaped fracture modules with different apertures of 1 mm, 3 mm, and 5 mm were, respectively, plugged. After assembling the device, it was heated to the phase transition temperature, and the temperature-activated liquid–solid phase transition plugging emulsion was pumped into the fractures from the drilling fluid kettle. After high-temperature response at 90 °C, within 3 h, plugging particles with a wide particle size distribution were in situ-generated in fractures with different apertures. Under the condition of continuously increasing pressure, the in situ self-generated plugging particles entered the deep fractures until the plugging was broken through, and the obtained maximum pressure was the plugging pressure-bearing capacity.

**Figure 24:**
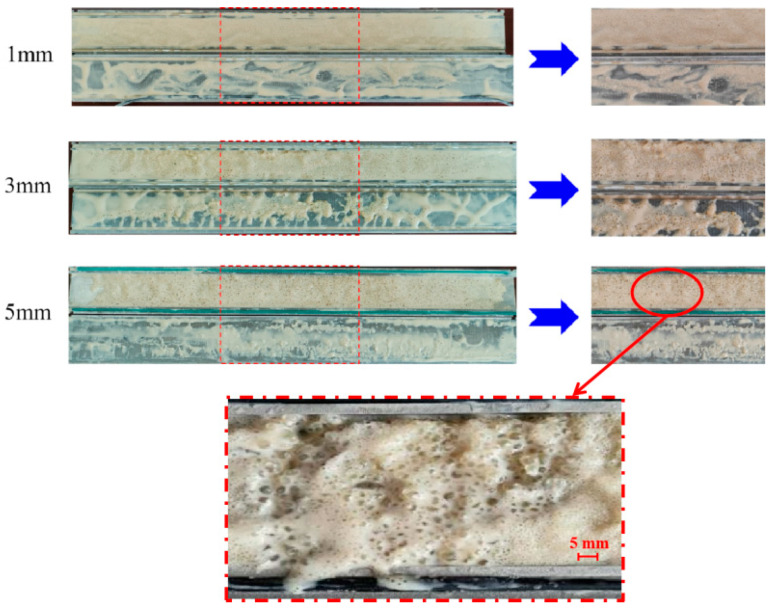
Sealing layers can be formed by particles in fractures with different openings, ranging from 1 to 5 mm.

After the experiment, the wedge-shaped fracture module was opened, as shown in Figure 24. The results show that the temperature-activated liquid–solid phase transition plugging emulsion, after high-temperature response at 90 °C, can in situ generate plugging particles with a wide particle size distribution in fractures with different apertures. Large particle size particles form bridges, medium particle size particles fill pores, and small particle size particles achieve tight plugging. After liquid–solid phase transition, the pressure-bearing capacity of the self-generated particles reaches 10 MPa without breakthrough. Using only one system, it exhibits good plugging performance for fractures with apertures of 1–5 mm, achieving adaptive plugging.

The primary innovation of this study lies in proposing an in situ self-adaptive phase-change plugging strategy that overcomes the poor adaptability of conventional bridging lost-circulation materials (LCMs) and addresses the technical challenge of severe mud losses in formations with unknown fracture apertures. A liquid–solid phase-change lost circulation system was constructed that remains fully liquid under surface conditions. The system comprises a phase-change fluid and a non-phase-change fluid, enabling effortless injection into fractures of varying widths and eliminating the strong size-matching dependence inherent in conventional LCMs. Upon exposure to down-hole high-temperature stimuli, the phase-change fluid crosslinks and agglomerates in situ into high-strength bridging particles with a broad particle size distribution, whereas the non-phase-change fluid remains liquid to preserve conductive channels. The proposed in situ self-adaptive phase-change plugging strategy significantly increases the success rate of single-stage lost circulation control, surmounts the limitations of conventional bridging LCMs (e.g., face plugging and re-loss), and provides a viable solution for severe mud losses in formations with unknown fracture apertures.

## 4. Conclusions

This study developed a temperature-activated liquid–solid phase transition plugging emulsion, and investigated its synthesis method, structural characterization, and performance evaluation, with a particular focus on the control factors affecting particle size distribution and fracture sealing efficiency. The main conclusions are as follows:

1. Based on thermosetting resin emulsification and high-temperature crosslinking coalescence the principles, a temperature-activated liquid–solid phase transition plugging emulsion was developed. The optimized formulation contains an emulsifier content of 8 wt.%, dispersant content of 0.16 wt.%, crosslinker content of 0.4 wt.%, distilled water content of 70 wt.%, viscosifier content of 0.4 wt.%, and nano-silica content of 2.0 wt.% (percentages relative to polymer resin content). The system generates particles with 1–5 mm size distribution, suitable for 80–120 °C loss formation, achieving complete curing within 140–180 min.
2. The system exhibits excellent injectability, rapidly forming impermeable sealing layers in loss zones. Under 60 MPa pressure, the aged plugging particles’ particle size degradation rate is small at 120 °C, 140 °C, 160 °C, and 180 °C, confirming superior thermal stability and compressive strength.
3. Using a single formulation, the plugging emulsion achieves 10 MPa breakthrough pressure resistance with self-adaptive sealing capability for 1–5 mm fractures. The in situ-generated particles maintain effective bridging–filling–sealing functions across varying fracture apertures.

## Figures and Tables

**Table 1:** Experimental reagents.

Reagents	Abbreviation	Manufacturer	Purity
Macromolecular resin	XP	Shanghai McLean Biochemical Technology Co., Ltd. (Shanghai, China)	AR
Emulsifier	CK-1		AR
Crosslinker	JL-1		AR
Dispersant	FS-1		AR
Viscosifier	ZN-1		LR
Nanomaterial	Nano-SiO_2_		AR

**Table 2:** Experimental instruments.

Serial Number	Experimental Instruments	Manufacturer
1	Electric-Heating Forced-Air-Drying Oven	Wuxi Marit Technology Co., Ltd. (Wuxi, China)
2	Analytical Balance	Shanghai Zhendu Electronic Technology Co., Ltd. (Shanghai, China)
3	Triple-Purpose Electronic Constant Temperature Water Tank	Shanghai Huayan Instrument Equipment Co., Ltd. (Shanghai, China)
4	JJ-1H Precision Force-Increasing Electric Stirrer	Shanghai Kexing Instrument Co., Ltd. (Shanghai, China)
5	Digital Display Building Material Pressure-Testing Machine	Jinan Tianchen Testing Machine Manufacturing Co., Ltd. (Jinan, China)
6	Fourier Transform Infrared Spectrometer	Bruker Corporation, Bremen, Germany
7	DMA Q800 Thermomechanical Analyzer	TA Instruments, New Castle, DE, USA
8	BX53 Optical Microscope	Olympus Optical Technology Co., Ltd., Tokyo, Japan
9	Microscopic Visualization Displacement Device	Jiangsu Tuochuang Scientific Research Instrument Co., Ltd., Jiangsu, China
10	ULTIM Max65 Scanning Electron Microscope	Thermo Fisher Scientific (Shanghai, China)
11	Standard Sieve	Shaoxing Shangyu Shengchao Instrument Equipment Co., Ltd., Shaoxing, China
12	HGRL-4A Aging Furnace	Chongqing Xindequan Petroleum Special Instrument Factory, Chongqing, China
13	Six-Speed Rotational Viscometer	Qingdao Shande Petroleum Instrument Co., Ltd., Qingdao, China
14	Variable-Frequency High-temperature Roller Heating Furnace	Qingdao Senxin Electromechanical Equipment Co., Ltd., Qingdao, China
15	High-Temperature And High-Pressure Dynamic Plugging Simulation Experimental Device	Hai’an Petroleum Scientific Research Instrument Co., Ltd., Hai’an, China

**Table 3:** The influence of different temperatures on the phase transition time of the plugging emulsion.

Serial Number	Temperature/°C	Phase Transition Time/Min
1	80	180
2	90	180
3	100	160
4	110	150
5	120	140

**Table 4:** The influence of nano-silica on compressive strength of plugging particles.

Temperature/°C	Without Nano-Silica	2 wt.% Nano-Silica
	D(90) Degradation Rate/%	D(90) Degradation Rate/%
120	5.25	3.07
140	7.14	4.11
160	8.03	5.06
180	10.84	5.41

Note: 2 wt.% was the mass fraction of resin.

**Table 5:** Main mesh number distribution of in situ self-generated plugging particles.

Mesh Number	Mass Fraction/%
3–8 Mesh	27.21
8–20 Mesh	65.06
20–40 Mesh	7.55
40–100 Mesh	0.18
Total amount	100.00

**Table 6:** D(90) value of plugging particles at different temperatures.

Phase Transition Temperature/°C	D(90)/mm
80	2.06
90	3.51
100	3.67
120	3.74

**Table 7:** Viscosity test of temperature-activated liquid–solid phase transition plugging emulsion.

Experimental Group	Apparent Viscosity/mPa·s	Plastic Viscosity/mPa·s	Yield Point/Pa
1	83.5	43.0	40.5
2	84.0	43.0	41.0

## Data Availability

The original contributions presented in this study are included in the article. Further inquiries can be directed to the corresponding author.
